# Supplementary material for: Magnetization dependent tunneling conductance of ferromagnetic barriers
Source: Nat Commun. 2021 Nov 18;12:6659. doi: 10.1038/s41467-021-26973-7 (PMC8602639; doi:10.1038/s41467-021-26973-7)
Supplement: Supplementary file 1 — Supplementary Information [file 41467_2021_26973_MOESM1_ESM.pdf]

Supplementary Information for "*Magnetization dependent tunneling conductance of ferromagnetic barriers*"

Zhe Wang,[1,2] Ignacio Gutiérrez-Lezama,[2,3] Dumitru Dumcenco,[2] Nicolas Ubrig,[2,3] Takashi Taniguchi,[4] Kenji Watanabe,[5] Enrico Giannini,[2] Marco Gibertini,[6,2] and Alberto F. Morpurgo[2,3]

[1]*MOE Key Laboratory for Nonequilibrium Synthesis and Modulation of Condensed Matter, School of Physics, Xi'an Jiaotong University, Xi'an,710049, China*
[2]*Department of Quantum Matter Physics, University of Geneva, 24 Quai Ernest Ansermet, CH-1211 Geneva, Switzerland*
[3]*Group of Applied Physics, University of Geneva, 24 Quai Ernest Ansermet, CH-1211 Geneva, Switzerland*
[4]*International Center for Materials Nanoarchitectonics,National Institute for Materials Science, 1-1 Namiki, Tsukuba, 305-0044, Japan*
[5]*Research Center for Functional Materials,National Institute for Materials Science, 1-1 Namiki, Tsukuba, 305-0044, Japan*
[6]*Dipartimento di Scienze Fisiche, Informatiche e Matematiche, University of Modena and Reggio Emilia, IT-41125 Modena, Italy*

Supplementary Note 1: Characterizations of bulk crystals

$CrBr_3$ single crystals were grown by chemical vapor transport [1] (see optical microscope images in supplementary Fig. **1**). As characterization of the grown crystals, we have performed X-ray diffraction (XRD) and Energy Dispersive X-ray spectroscopy (EDX) to confirm the expected crystal structure and composition. XRD characterization was done on a Bruker D8 Advance system with a Cu-$K_{\alpha 1}$ monochromator. It demonstrated the expected crystal structure, a high level of crystallinity of the samples and the [00l] orientation of the platelets. EDX analysis was performed with an Oxford SDD X-MaxN80 probe in a JEOL JSM 7600 F electron microscope, and confirmed the chemical composition of Cr:Br being 1:3 and the high homogeneity of the $CrBr_3$ crystals.

As further characterization, we also performed magnetization measurements on a crystal of 2.1mg with a MPMS3 SQUID magnetometer from Quantum Design. The temperature dependence of the magnetization shows a clear paramagnetic to ferromagnetic transition (Fig.1b of the main text), and gives a Curie temperature $T_c \approx 32K$ (as estimated from the minimum of dM/dT, see inset of Fig.1b of the main text), in agreement with previous reports [2–5]. Isothermal magnetization measurements at 2 K (supplementary Fig.1) show that bulk $CrBr_3$ crystals exhibit the behavior typical of soft ferromagnets, with virtually negligible magnetic hysteresis upon sweeping the applied magnetic field. The saturation field is 0.33 Tesla for $H//c$-axis and 0.67 Tesla for $H \perp c$-axis, confirming that the easy axis of the $CrBr_3$ cyrstals is along c-axis. The saturation moment at 2 K is estimated to be $M_s \approx 3\mu_B/Cr$, consistently with previous reports [6,7].

Supplementary Note 2: Data of $N = 8$ layer device

To show the reproducibility of our results and considerations, we show the data and the analysis done for the N=8 layer device in Supplementary Fig. **4**, which exhibits an identical behavior as the one of the N=7 device discussed in the main text.

Supplementary Note 3: Extracting magnetization of thin flakes from tunneling conductance

In the main text, we have demonstrated that tunneling magnetoconductance of $CrBr_3$ barriers is entirely determined by the material magnetization. This finding implies that detailed information about the behavior of the magnetization of atomically thin crystals can be extracted directly from tunneling conductance measurements, providing a new method to investigate magnetic properties of 2D ferromagnetic insulators. Here we elaborate on a few specific points that have been mentioned in the main text.

First, in the main text we mentioned that –for small magnetization values– the magnetococnductance is proportional to the square of the magnetization M. This is shown in supplementary Fig. **5**, in which the magnetocondcutance is plotted as a function of M^2. It is clearly visible that for small M the dependence is linear. This experimental observation is useful to extract information about $M(H,T)$ close to T_c, where M is small, by simply taking the square root of magnetoconductance. As an example, in the main text we have mentioned that this quadratic dependence

of the magnetocconductance on magnetization can be extremely useful to make a detailed quantitative analysis of critical exponents of 2D ferromagnets, since magnetoconductance measurements are experimentally particularly simple to perform.

We also note that the linear dependence of magnetoconductance on the square of the magnetization can be straightforwardly understood within the model introduced in the main text, based on the Fowler-Nordheim expression for the tunneling current. For tunneling through a ferromagnetic insulator in the Fowler-Nordheim regime, the height of the tunnel barrier is different for spin-up and spin-down electrons, as soon as the magnetization is non-zero. We can then write:

$$G = G^{\uparrow} + G^{\downarrow} = A\frac{V}{\phi_{\uparrow}}e^{\frac{-8\pi d\sqrt{2m^*}\phi_{\uparrow}^{3/2}}{3heV}} + A\frac{V}{\phi_{\downarrow}}e^{\frac{-8\pi d\sqrt{2m^*}\phi_{\downarrow}^{3/2}}{3heV}}, \tag{1}$$

where A is a constant related to the device geometry, m^* is the effective mass, h is Planck's constant, e the modulus of the electron charge, and $\phi_{\uparrow}$ and $\phi_{\downarrow}$ and the tunneling barrier for spin-up and spin-down electrons respectively. The tunneling conductance is determined by the magnetization M through the relation $\phi_{\uparrow,\downarrow} = \phi_{B0} \pm \gamma M$, where ϕ_{B0} is the tunneling barrier at $M = 0$. If γM is much smaller than ϕ_{B0}, then we can take $\epsilon = \gamma M/\phi_{B0}$ to be a small quantity and perform a Taylor expansion (here below we denote $\beta \equiv \frac{8\pi d\sqrt{2m^*}}{3he}$):

$$\begin{aligned}
G &= \frac{AV}{\phi_{B0}(1+\epsilon)}e^{-\beta\phi_{B0}^{3/2}(1+\epsilon)^{3/2}/V} + \frac{AV}{\phi_{B0}(1-\epsilon)}e^{-\beta\phi_{B0}^{3/2}(1-\epsilon)^{3/2}/V} \\
&= \frac{AV}{\phi_{B0}}\left[\left(1-\epsilon+\epsilon^2+\dots\right)e^{-\beta\phi_0^{3/2}\left(1+3/2\epsilon+3/8\epsilon^2+\dots\right)/V} + \left(1+\epsilon+\epsilon^2+\dots\right)e^{-\beta\phi_{B0}^{3/2}\left(1-3/2\epsilon+3/8\epsilon^2+\dots\right)/V}\right] \\
&\approx \frac{AV}{\phi_{B0}}e^{-\beta\phi_{B0}^{3/2}(1+3/8\epsilon^2)/V}\left[\left(1-\epsilon+\epsilon^2\right)e^{-3\epsilon\beta\phi_{B0}^{3/2}/(2V)} + \left(1+\epsilon+\epsilon^2\right)e^{3\epsilon\beta\phi_{B0}^{3/2}/(2V)}\right] \\
&= \frac{2AV}{\phi_{B0}}e^{-\beta\phi_{B0}^{3/2}(1+3/8\epsilon^2)/V}\left[\left(1+\epsilon^2\right)\cosh\left(\frac{3\epsilon\beta\phi_{B0}^{3/2}}{2V}\right) + \epsilon\sinh\left(\frac{3\epsilon\beta\phi_{B0}^{3/2}}{2V}\right)\right]
\end{aligned} \tag{2}$$

For T close to T_c and small magnetic field, the equation above can be further approximated as

$$G(T,H) \approx G_0 + \frac{\gamma^2 G_0}{\phi_{B0}^2}\left[1 + \frac{9\beta\phi_{B0}^{3/2}}{8V}\left(\frac{\beta\phi_{B0}^{3/2}}{V}+1\right)\right]M^2(H,T) \tag{3}$$

where G_0 is the conductance at $M = 0$.

Finally, we can also extract a full map of $M(H,T)$ by using Eq. (1) above to calculate the magnetoconductance, equate it to the experimentally measured value for each value of H and T, and solve the equation numerically. Specifically, we have

$$\delta G = \frac{G - G(M=0)}{G(M=0)} = [\frac{1}{(\phi_{B0}+\gamma M)}e^{\frac{-\beta(\phi_{B0}+\gamma M)^{3/2}}{V}} + \frac{1}{(\phi_{B0}-\gamma M)}e^{\frac{-\beta(\phi_{B0}-\gamma M)^{3/2}}{V}}]/[\frac{2}{\phi_{B0}}e^{\frac{-\beta\phi_{B0}^{3/2}}{V}}] - 1, \tag{4}$$

and we equate it to the tunneling magnetoconductance measured in the experiments:

$$\delta G = \begin{cases} \frac{G(H,T)-G(0,T)}{G(0,T)} & T \geq T_c \\ \frac{G(H,T)-G(0,T_c)}{G(0,T_c)} & T < T_c \end{cases}$$

Equation (4) is solved for the quantity γM. Since γ is constant, we obtain the full map of $M(H,T)$ in unit of M_s, under the assumption that the saturation magnetization is reached at 2K and 3 Tesla. The result of the procedure is shown in supplementary Fig. **6**. We have checked that the value of electron effective mass in $CrBr_3$ has virtually no influence on the final result (the difference in M/M_s obtained after solving the equations for $m^* = m_0$ and $m^* = 10m_0$ is less than 0.01%).

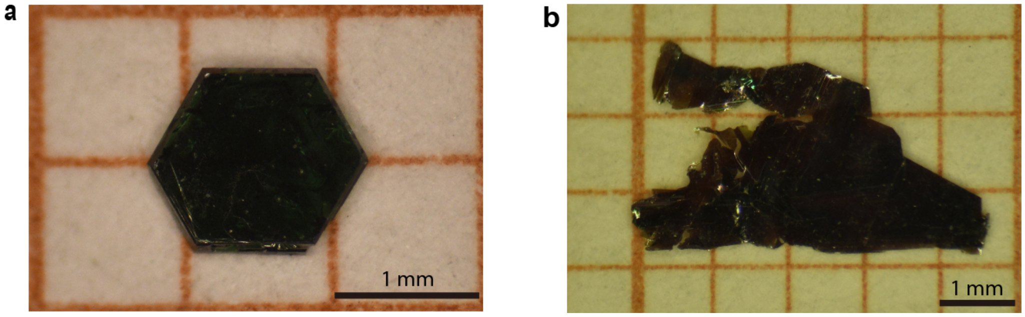

Supplementary Fig. 1. **Optical images of $CrBr_3$ crystals.** **a**: Optical microscope image of a $CrBr_3$ crystal grown by vapor phase transport: the hexagonal shape is indicative of the single crystal nature. **b** Vapor phase transport also results in rather large poly-crystals formed by multiple individual single crystals of different orientation and size merged together. The scale bar in both panels is 1 mm.

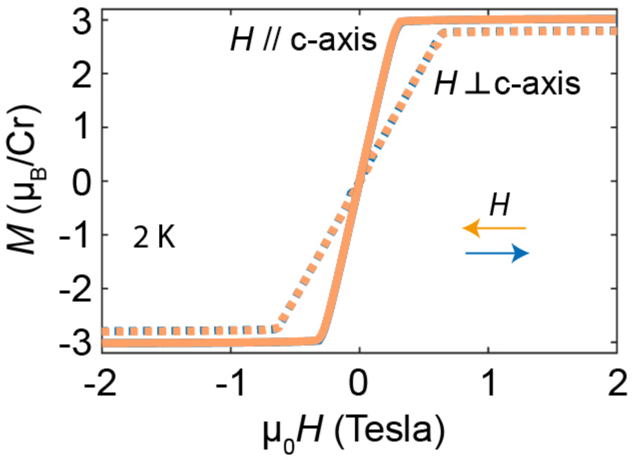

Supplementary Fig. 2. **Magnetic field dependence of bulk magnetization at 2 K.** The solid lines represent the magnetization measured with the magnetic field applied parallel to the c-axis of $CrBr_3$ crystals (i.e., the field is perpendicular to the layers), and the dashed lines with the field applied perpendicular to the c-axis (i.e., the field is applied in the plane).

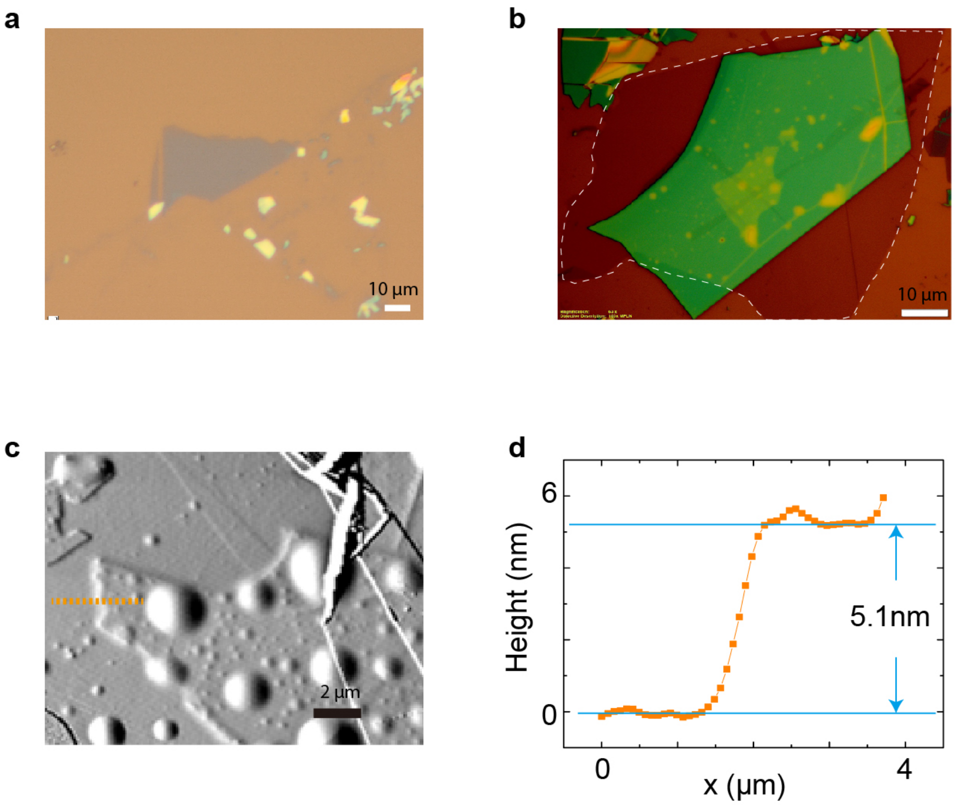

Supplementary Fig. 3. **Images of the 7-layers device.** **a**: Optical microscope image of the 7-layer $CrBr_3$ used for the realization of one of the devices discussed in this paper, after exfoliation on a SiO_2 substrate. **b**: Optical microscope image of the encapsulated tunnel junction device formed by hBN/graphite/$CrBr_3$/graphite/hBN layers, prior to the deposition of the metal contacts. The darker green large crystal is the top hBN layer; the dashed white line marks the edges of the bottom hBN layer; two crossed graphite ribbons are also faintly visible, positioned on opposite sides of the $CrBr_3$ layer, which is the light green region in the middle of the image. **c**: Atomic force microscope image of $CrBr_3$ after encapsulation. **d**: Height profile of the $CrBr_3$ flake measured along the dotted line in panel (c). The step height of ~ 5.1nm indicates the $CrBr_3$ is 7 layers.

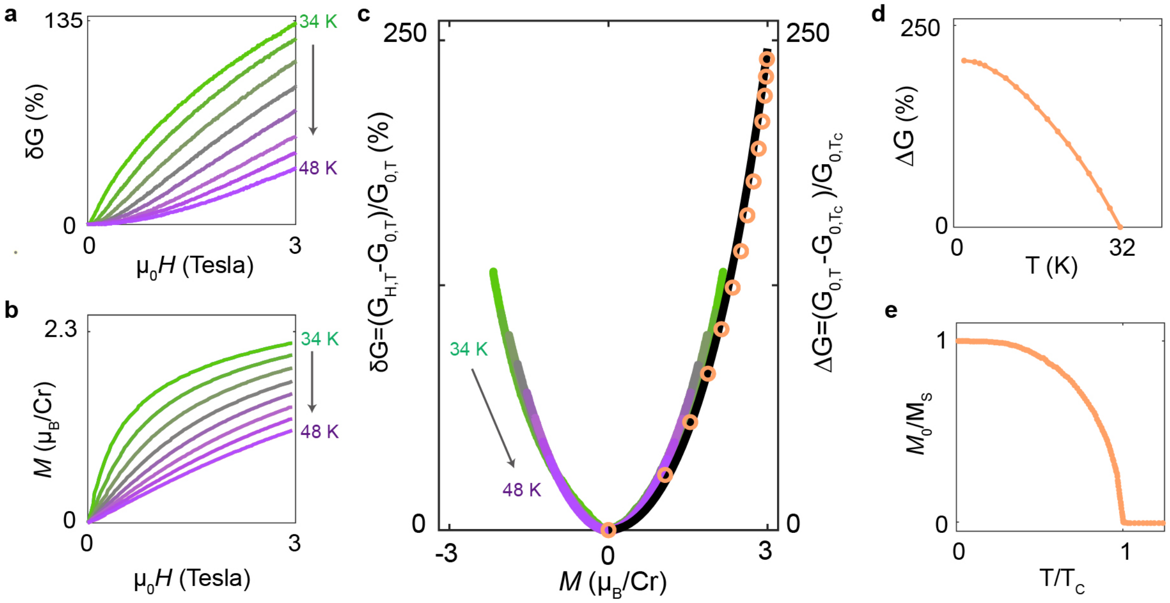

Supplementary Fig. 4. **Magnetization dependence of the conductance in the 8-layer device.** **a**: Tunneling magnetoconductance measured on the 8-layer device at different temperatures in the range 34-48 K (in steps of 2 K). **b**: Magnetic field dependence of the magnetization of bulk $CrBr_3$ crystals. **c**: Tunneling magneto-conductance of the 8-layer device plotted as a function of magnetization. Colored lines represent the magnetoconductance measured above T_c, and the orange empty circles represent the relative change in conductance originating from the spontaneous magnetization in the ferromagnetic state of $CrBr_3$, for $T < T_c$, as obtained from the data plotted in panels **d** and **e**. The black line is the best fit based on the Fowler-Nordheim expression for the magnetoconductance, from which we extract an energy splitting between the up and down spins of 113 meV for $m^* = m_0$ (almost the same as the one obtained from the 7-layer device discussed in the main text). In panels **a-c** curves of the same color correspond to data measured at the same temperature, indicated in panel **a**. **d**: Temperature dependence of the relative change in conductance due to the increase of the spontaneous magnetization in $CrBr_3$ upon lowering temperature. **e**: Spontaneous magnetization calculated with the XXZ model with anisotropic exchange interactions. The data in this figure show that the 8-layer device exhibits a behavior that is identical to that of the 7-layer device discussed in the main text. The only difference between the two devices is the absolute value of magnetoconductance due to the different values of applied voltage (and correspondingly of electric field; indeed, the strong non-linearity of the $I - V$ tunneling curve implies that the exact values of conductance depend on the applied bias).

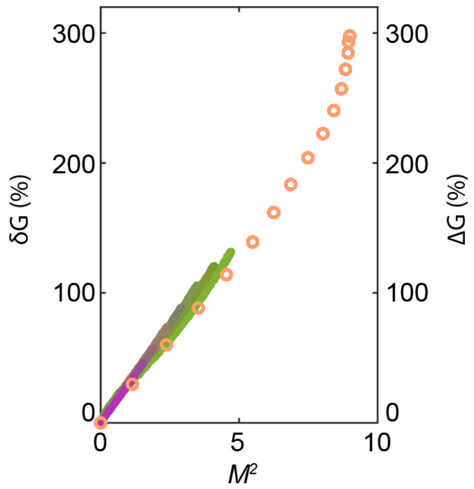

Supplementary Fig. 5. **Linear dependence of the magnetoconductance on the square of the magnetization.** Solid lines represent the magnetoconductance δG measured at different values of temperature $T > T_c$; the empty orange dots represent the relative change in conductance ΔG upon varying $T < T_c$ (at $H = 0$), due to the increase of the spontaneous magnetization M in the ferromagnetic state. For small values of magnetization, both quantities –already discussed in the main text– show a linear dependence when plotted as a function of M^2. As mentioned in the main text, this quadratic dependence can be used to investigate the critical behavior of $M(H,T)$ close to T_c (and in particular the critical exponents) from simple conductance measurements.

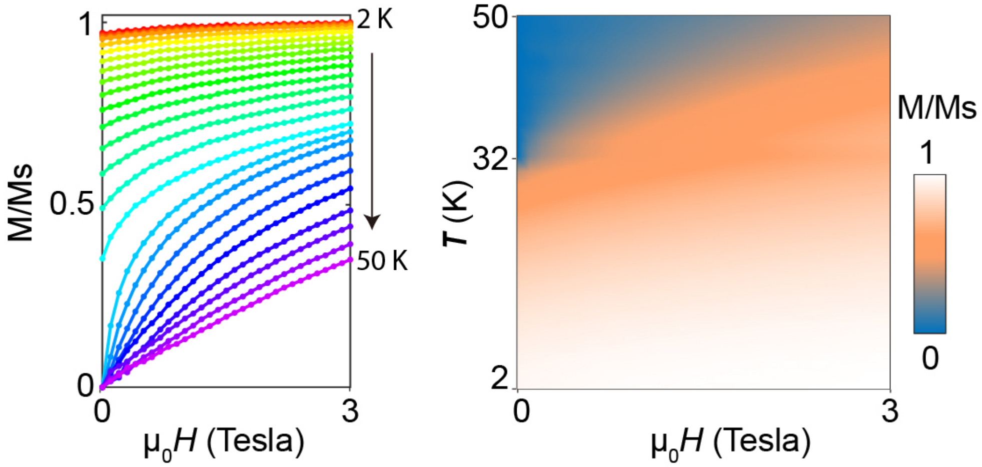

Supplementary Fig. 6. **2D map of magnetization determined from tunneling conductance.** As we have demonstrated in the main text directly from the exerimental data (i.e., without the need to make any theoretical assumption), the tunneling magnetoconductance of $CrBr_3$ barriers is solely dependent on $M(H,T)$. If in addition we also use the theoretical expression based on the FN tunneling model –which we have shown to excellently reproduce the experimental observations– we can extract the magnetization of the $CrBr_3$ layer by numerically solving equation 4 in the supplementary information, as discussed above. The result is shown in the two panels in this figure. The left panel is the magnetization (normalized to the saturatiuon magnetization) at different values of T between 2 K and 50 K, as a function of applied magnetic field. The right panel shows the same data as a color plot (it is assumed that the magnetization at 2 K and 3 T corresponds to the saturation value M_s).

[1] Dumcenco, D. & Giannini, E. Growth of van der Waals magnetic semiconductor materials. *Journal of Crystal Growth* **548,** 125799 (2020).

[2] Jennings, L. & Hansen, W. Heat capacity of $CrBr_3$ from 14 to 360 ° k. *Physical Review* **139,** A1694 (1965).

[3] Ho, J. T. & Litster, J. D. Divergences of the magnetic properties of $CrBr_3$ near the critical point. *Journal of Applied Physics* **40,** 1270–1271 (1969).

[4] Samuelsen, E., Silberglitt, R., Shirane, G. & Remeika, J. Spin waves in ferromagnetic $CrBr_3$ studied by inelastic neutron scattering. *Physical Review B* **3,** 157 (1971).

[5] Yu, X. *et al.* Large magnetocaloric effect in van der Waals crystal $CrBr_3$. *Frontiers of Physics* **14,** 1–5 (2019).

[6] Tsubokawa, I. On the magnetic properties of a $CrBr_3$ single crystal. *Journal of the Physical Society of Japan* **15,** 1664–1668 (1960).

[7] McGuire, M. A. Crystal and magnetic structures in layered, transition metal dihalides and trihalides. *Crystals* **7,** 121 (2017).
